# Green Composites Based on Unsaturated Polyester Resin from Recycled Poly(Ethylene Terephthalate) with Wood Flour as Filler—Synthesis, Characterization and Aging Effect

**DOI:** 10.3390/polym12122966

**Published:** 2020-12-11

**Authors:** Przemysław Pączkowski, Andrzej Puszka, Barbara Gawdzik

**Affiliations:** Department of Polymer Chemistry, Institute of Chemical Sciences, Faculty of Chemistry, Maria Curie-Sklodowska University in Lublin, Gliniana 33, 20-614 Lublin, Poland; andrzej.puszka@umcs.pl (A.P.); barbara.gawdzik@umcs.pl (B.G.)

**Keywords:** unsaturated polyester resins, recycled PET, wood flour, dibenzylideneacetone, green composites, accelerated aging test

## Abstract

The paper investigates the synthesis of green composites and their properties before and after the laboratory accelerated aging tests. Materials were made of unsaturated polyester resins (UPRs) based on recycled poly(ethylene terephthalate) (PET) and wood flour (WF). The effect of dibenzylideneacetone (dba) addition on mechanical and thermomechanical properties was also determined. Green composites were obtained using environment friendly polymeric cobalt as an accelerator. Before and after exposition to the xenon lamp radiation, the UPRs physically modified by WF were characterized only by a greater flexural modulus compared with the analogous composites based on the pure resin. Addition of dba caused the increase of flexural modulus, flexural strength, strain at break and mechanical loss factor compared to the non-modified material. After aging only the last mentioned parameter took on lower values compared to the pure resin analogues.

## 1. Introduction

In the last decades due to growing depletion of resources and global awareness of the environment on the Earth, an increasing interest to economically and environmentally friendly biobased materials and recycling the raw material wastes was observed. The composites made from synthetic polymeric resin and natural organic fillers such as wood flour, wood fibers or agricultural wastes are often used [1,2,3,4,5]. Unsaturated polyester resins are the most common in preparing composites, comprising 80% excess of all thermoset resins [6]. Recently, unsaturated polyester resin (UPR) from recycled poly(ethylene terephthalate) (PET) has drawn the attention of many researchers owing to the cost-effective product for polymeric composites [7,8,9,10].

Significant importance of wood as reinforcement for the polymeric composite is due to the fact that the biofiller has the advantages such as high-specific strength, low cost, low-density, biodegradability, low water absorption and thickness swelling. The positive effect of using these waste products from the wood industry is ecofriendliness. Softwood flour is a cellulose-containing material and its chemical composition is as follows, cellulose (40–44%), hemicellulose (25–29%) and lignin (25–31%) [11]. The combination of cellulose and hemicellulose is called holocellulose and usually accounts for 65–70% of the dry wood weight. Those mentioned major carbohydrate portions of wood are made up of simple sugars mainly D-glucose, D-mannose, D-galactose, D-xylose, L-arabinose and D-glucuronic acid but their precise composition depend on the tree species, geographic location, climate and soil condition [12]. Fillers are added into the polymer matrix to improve their properties. According to Marcovich et al. [2] polymers with particulate fillers or short fiber reinforcements are generally well processable. Besides, these products are completely degradable at the end of their utility period by means of biodegradation and/or combustion. However, the main disadvantages of using such fibers in the composites are their hygroscopicity and difficulty in achieving acceptable levels of dispersion in the polymer matrix. One approach to reduce hygroscopicity is esterification of the wood component. Modification of wood with maleic anhydride improves its mechanical properties and induces stability while reducing water absorption [13]. The studies by Rahman et al. [14] show that the incorporation of maleic anhydride increases the flexural strength and flexural modulus of composites compared to the ungrafted ones.

Unfortunately, the products of unsaturated polyester resins undergo surface degradation during the use, which was confirmed by the aging tests carried out by Zhao et al. [15]. In the artificial weathering environment ester bonds in the polymeric structure broke, the gloss decreased with the increasing exposure time due to the increase of surface roughness, and the surface turned darker. They postulated to add appropriate supporters into the formula to prevent the interactions of light and water on the unsaturated polyester resins when they are used under the outdoor conditions.

In this study, an attempt was made to evaluate the mechanical and thermomechanical properties of the green composites from the unsaturated polyester resin based on the recycled PET, which were reinforced with softwood flour as a biofiller. The materials were characterized before and after the laboratory accelerated aging test. The effect of the addition of dibenzylideneacetone acting as the UV absorber on the properties of wood–resin composites was also investigated [16,17]. The purpose of this study was to determine effect of dibenzylideneacetone addition on the resin aging.

## 2. Materials and Methods

### 2.1. Chemicals

The estromal orthophthalic resin based on recycled PET (LERG, Pustków, Poland) is a bluish-green colored liquid. The properties provided by the manufacturer are acidic number (13.4 mg KOH g^−1^; PN-EN ISO 2114:2005), viscosity (356 mPas at 23 °C; PN-EN ISO 12058-1:2018), non-volatile content (61.2%; PL-W-53) and reactivity factor (1.53; PN-EN ISO 584:2002).

For curing of UPR, Luperox DHD-9 (2-butanone peroxide solution) (Sigma Aldrich, St. Louis, MO, USA), and 4% polymeric cobalt solution (Department of Polymer Chemistry, Maria Curie-Sklodowska University in Lublin, Poland) were used. Additionally, dibenzylideneacetone was synthesized in the Department of Polymer Chemistry, Maria Curie-Sklodowska University in Lublin, Poland. For physical modification of unsaturated polyester resin, spruce and fir softwoods providing the raw material JELUXYL WEHO 100/f and 120/f (JELU-WERK, Rosenberg-Ludwigsmühle, Germany) were used. Sieves fractions from Alpine air draft sieve were: 75 μm (35%), 100 μm (20%) and 180 μm (traces).

### 2.2. Preparation of Green Composites

The unsaturated polyester resin (UPR) was preaccelerated with 0.25 wt% of the 4% polymeric cobalt solution and mixed for 5 min to obtain homogeneity. This procedure was repeated at the point where the suitable amount of wood flour (WF) or dibenzylideneacetone (dba) was added to the pure resin. To this mixture, 1.1 wt% of Luperox DHD-9 as an initiator was added. All ingredients amount was calculated for the pure resin. The prepared mixtures were poured into the cuboid-shaped molds with the dimensions 145 mm × 100 mm × 4 mm. Curing was conducted in the molds at room temperature for 24 h and then they were placed in an oven for 10 h at 80 °C for the additional post-curing process. The same procedure was applied for the pure resin and composites with WF or dba. The compositions of the prepared blends are summarized in Table 1.

#### Preparation of Samples

In order to determine the mechanical and thermomechanical properties of the obtained composites, the samples were prepared in an appropriate manner. The CNC-milling machine MFG 8037P (Ergwind, Gdańsk, Poland) was used to cut off a rectangular bar with the dimensions 80 mm × 10 mm × 4 mm. The samples were exposed to artificial aging in a simulator where the irradiation source of this apparatus is composed of a xenon arc lamp emitting radiation similar to the natural sunlight. An additional sample, which was not used in the accelerated aging studies, served as a reference material.

### 2.3. Research Methods

The accelerated aging test was taken using a Xenon Arc Lamp simulator Atlas Xenotest Alpha+ (Chicago, IL, USA). The irradiation source of this apparatus is composed of a centrally located xenon arc lamp in the test chamber, which emits radiation similar to the natural sunlight. An irradiance of 60 W/m^2^, daylight filter system, chamber temperature of 38 °C, black standard temperature of 65 °C, relative humidity of 50%, spray off/on periods (18 min spray on and 102 min off) and time of 1000 h were used as the environmental conditions and degradation times for the accelerated aging process. The above-mentioned parameters mimic the typical weather conditions to which materials prepared for indoor spaces might be exposed. The test procedure is according to the following standard (PN-EN ISO 4892-2:2013).

The attenuated total reflectance Fourier transform infrared (FT-IR/ATR) spectra were obtained with a Bruker TENSOR 27 spectrometer with a diamond crystal (Ettlingen, Germany). The spectra were gathered from 600 to 4000 cm^−1^ with 32 scans per spectrum at a resolution of 4 cm^−1^.

Mechanical studies were carried out using the mechanical testing machine, ZwickRoell Z010 from ZWICK GmbH Co (Ulm, Germany). With a three-point bending test, the 80 mm × 10 mm × 4 mm sample profiles were used. The span was 64 mm, whereas the bending speed was 5 mm min^−1^. The final result was the arithmetic averaging of five measurements before and after the accelerated aging test.

Thermomechanical properties of materials were determined using the dynamic mechanical analyzer (DMA) Q800 from TA Instruments (New Castle, NY, USA) equipped with a dual-cantilever device. The samples of 65 mm × 10 mm × 4 mm dimension before and after the accelerated aging, were tested. The temperature scanning from 0 to 200 °C with a constant heating rate of 3 °C min^−1^ at a sinusoidal distortion of 10 µm amplitude and 1 Hz frequency was conducted. The glass-transition temperature, mechanical loss factor, values of storage modulus and loss modulus were determined.

Hardness was determined using a Barcol impressor hardness tester GYZJ 934-1 from Barber-Colman Company (Loves Park, IL, USA). The final result was the mean value of ten measurements before and after the accelerated aging test.

Gloss measurements were made using the triple-angle gloss meter, Zehntner ZGM 1110 from Zehntner GmbH Testing Instruments (Sissach, Switzerland). This device works simultaneously in the one in the three geometric units. Its incidence angle values at 20°, 60° and 85° correspond with a high gloss to matte surface. Gloss standard: 20° (86.8 GU), 60° (93.4 GU) and 85° (99.7 GU).

## 3. Results and Discussion

**Figure 1:**
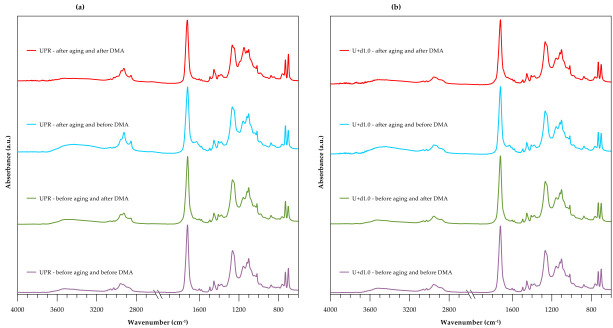
FT-IR/ATR spectra of the composites before and after the aging test and dynamic mechanical analyzer (DMA). (**a**) Pure resin and (**b**) resin with dba.

In Figure 1 the FT-IR/ATR spectra of composites based on the pure unsaturated polyester resin from recycled PET before and after the accelerated aging test and DMA are presented.

The characteristic peak in the spectra at 1719 cm^−1^ corresponds to C=O of the ester group. The peaks at 1260 and 1124 cm^−1^ are assigned to the asymmetric and symmetric C–O–C stretching vibrations of aromatic ester. The O-H stretching mode from the glycol end group in the polyester chain exists in the region about 3600 cm^−1^. The C-H stretching absorption peaks appear in the region from 3000 to 2700 cm^−1^. The peaks at 1615 (C=C), 1578 and 1505 cm^−1^ (C–C stretch) were associated with the phenyl ring and 1408 (C–C stretch), 1102 and 1018 (in plane bend), 983 and 875 (out of plane bend), 848 (ring breathing) and 794 and 730 cm^−1^ (out of plane bend). The family bands originating from the CH_2_ scissoring (1453 cm^−1^), wagging (1378 and 1339 cm^−1^), twisting (overlapping at 1250 cm^−1^) and rocking (898 cm^−1^) modes are also visible in the spectra.

Some bands can be used to differentiate the trans and gauche rotational isomers, characteristic of the crystalline and amorphous regions in PET. The peaks at 1339 and 1378 cm^−1^ represent the fraction of the glycol segment in the trans and gauche conformations, respectively [18,19]. According to Figure 1, the gauche conformation responsible for amorphous content in PET occurred to a much greater extent.

After the accelerated aging test in the Atlas simulator, the broad band at 3435 cm^−1^ corresponding to the OH group appeared. Additionally, the peak at 1628 cm^−1^ confirmed the presence of hydroxyl groups derived from the water molecule. This assumption was confirmed by the spectrum for the composites after DMA in which the bands from the OH groups of water disappeared. This is because the material was heated to 200 °C during the test and water present in the composite evaporated. In addition, the intensities of hydroxyl, C–O–C and methyl absorption peaks insignificantly decreased, which is attributed to degradation of the polyester chains. 

**Figure 2:**
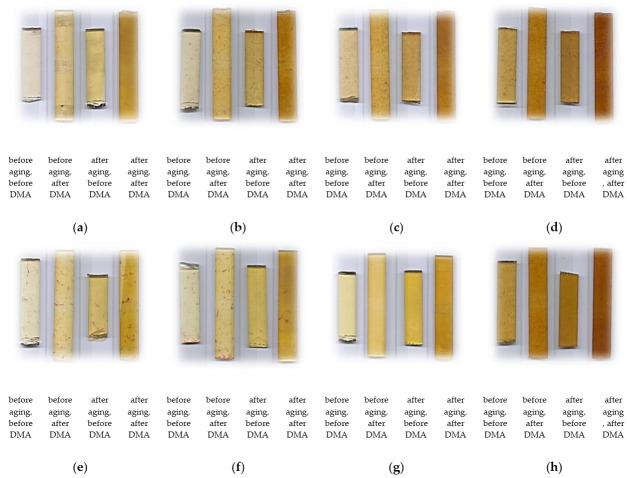
Samples before and after accelerated aging test and DMA. (**a**) Unsaturated polyester resin (UPR); (**b**) U+W1; (**c**) U+W2; (**d**) U+W5; (**e**) U+d0.2; (**f**) U+d0.5; (**g**) U+d1.0 and (**h**) U+W5+d1.0.

Figure 2 shows that the appearances of the samples changed with time, becoming more yellow by visual inspection in the course of ageing. There are many reasons for the color change. In this paper the polymer cobalt solution was used as an accelerator, its ions took part in the reaction and the surfaces of the samples ultimately appeared yellow. According to Sampers et al. [20] the cobalt-complexes absorb UV radiation and this absorption plays a significant role in the mechanism of discoloration of samples cured in the presence of cobalt accelerator The yellow hue also appeared when dba was used because of its characteristic bright-yellow color.

**Figure 3:**
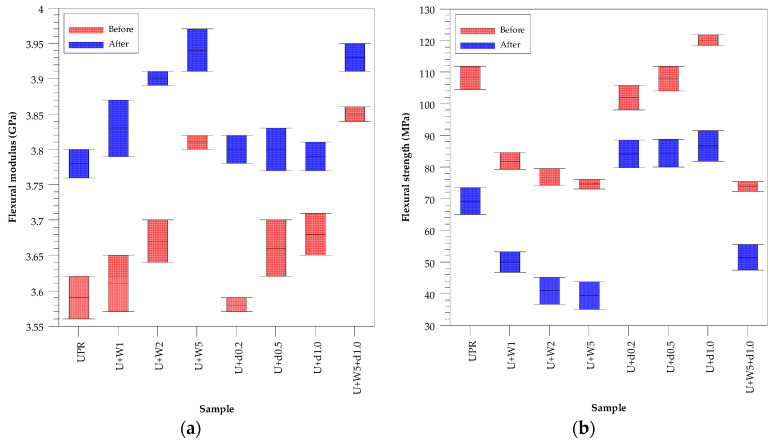

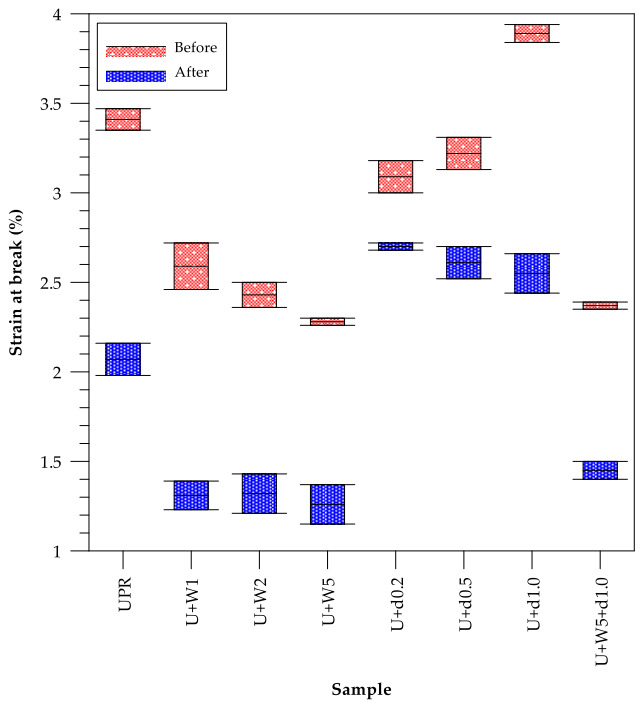
Mechanical data for pure UPR and composites before and after the accelerated aging test. (**a**) Flexural modulus; (**b**) flexural strength and (**c**) strain at break.

**Figure 4:**
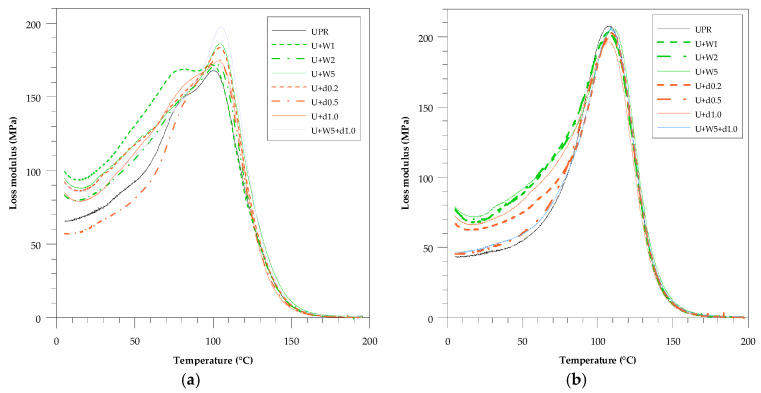
Loss modulus (E”) vs. temperature for pure UPR and green composites with wood flour (WF) and dba. (**a**) Before the accelerated aging test and (**b**) after the accelerated aging test.

**Figure 5:**
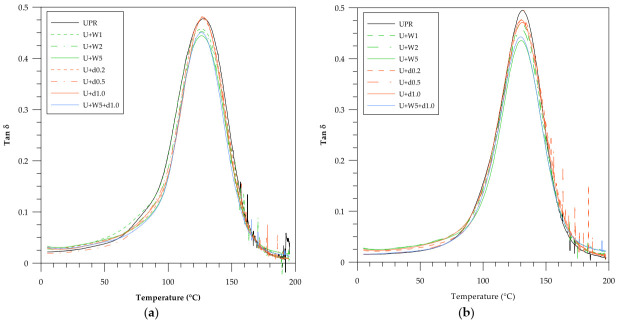
Mechanical loss factor (tan δ_max_) vs. temperature for pure UPR and green composites with WF and dba. (**a**) Before the accelerated aging test and (**b**) after the accelerated aging test.

The mechanical and thermomechanical properties of pure unsaturated polyester resin (UPR) and green composites containing wood flour (WF) and dibenzylideneacetone (dba) were determined. The results before and after the accelerated aging test of flexural modulus, flexural strength and strain at break are presented graphically in Figure 3, Figure 4 and Figure 5 whereas the numerical data are collected in Table 2, Table 3 and Table 4.

Wood is hydrophilic in nature, and the polymeric resin matrix is hydrophobic in nature. An important issue is that wood–resin composites are made up from two incompatible components and phases and their interactions at the interface, which can affect directly the strength properties. In the early stages of the ageing period, the polyester undergoes post-cure and physical ageing, whereas after a longer ageing period it undergoes a degree of degradation under the synergistic impact of ultraviolet radiation, high temperature and oxygen. The main influencing factor leading to the failure of the polyester is UV irradiation [21].

The incorporation of wood flour into the resin increased the flexural modulus from 3.59 to 3.81 GPa (Table 2). The flexural strength, strain at break and mechanical loss factor of green composites decrease 108–75 MPa, 3.41–2.28% and 0.4579–0.4227, respectively. The situation was diametrically different when dibenzylideneacetone was added to the resin. Using 1% addition of dba, the materials were characterized by higher values of all mentioned properties. 

After aging, the flexural modulus of the starting resin and composites with wood flour and dba increased (Table 3). In turn, their flexural strength and strain at break decreased significantly.

According to Table 4, the glass transition temperature of all green composites was larger than that for the pure resin (100 °C). With the increasing wood flour amount in the material, an increase of T_g_ from 101 to 105 °C was observed. The addition of dba to the resin caused an insignificant growth of this parameter. After the accelerated aging their values were 2–3 °C higher.

The effect of dba addition on the properties of the resin is shown in Figure 3. Dibenzylideneacetone is an ingredient absorbing UV radiation from the Sun, which is used to protect utility products [22]. Comparing the materials U+W5 and U+W5+d1.0 after accelerated aging, one can see that the latter was characterized by higher values of flexural strength and strain at break.

In Figure 4 and Figure 5 relationships between loss modulus versus temperature and mechanical loss factor versus temperature are presented. When comparing the curve before (a) and after (b) the accelerated aging test the main observation is the formation of a curve with a more Gaussian shape.

The width of the tan δ peak reflects the sample heterogeneity. A narrow peak indicates its homogeneous structure. Before the accelerated aging test (a) the composites containing wood flour were characterized by lower values of the mechanical loss factor than those based on pure resin (UPR). The situation is diametrically contrary in the case when the addition of dba to the resin increased the aforementioned parameter. After the accelerated aging test (b) the peak of the curve became narrower. The presence of dba in the sample U+W5+d1.0 caused the material to possess a higher value of tan δ than that of the U+W5 material. 

The storage modulus in the glassy, transition and rubbery regions characterize the viscoelastic behavior [23]. In the first region the material was very hard and rigid solid, as against the stiffness increase in the remaining region. After the accelerated aging test, the transition region decreased at the expense of the glassy region. The shape of the curve in the transition region was sharper (faster decline). The material exposed to the xenon lamp and higher temperature was post-cured and thus became hard.

It is generally known that fillers increase the hardness of materials based on the unsaturated polyester resin [24,25]. As we observed (Table 2 and Table 3), the values of the Barcol hardness increased from 36.0 to 45.2 after the strengthening of unsaturated polyester resin with wood flour. The presence of the filler reduced the brittleness and increased the resistance of the unsaturated polyester composites to deformations and it caused stronger interactions between atoms or molecules. As a result material hardness increased and thus its resistance to scratch grew. The addition of dba makes the surface of the composite even harder (48.4–52.0).

After aging the greatest hardness values were found for the pure resin (56.4) whereas the addition of wood flour caused a decrease of hardness due to the beginning of the degradation process. Values of hardness for composites with increasing content of dba were insignificantly lower. Sample U+W5+d1, containing 1% of dba had hardness 55.1 °B. The use of dba allows obtaining a durable, stable material in which changes proceed much more slowly.

The effect of the accelerated aging to the surface gloss is presented in Table 5. The 60° geometry can be used for all materials, but for very high gloss, the measuring method with 20° geometry is recommended. Before aging it can be observed that with the addition of wood flour, the gloss value decreased. All the samples were characterized as high gloss materials because their value at 60° geometry was greater than 70 GU and at 20° the situation was similar. After aging the pure resin is a medium gloss composite (50.2 GU). Gloss values of the composites with WF were lower than 70 GU. The addition of dba to the composite with the wood flour (U+W5+d1.0) allowed us to obtain high gloss material after accelerated weathering.

Gloss is an important parameter characterizing the material surface. When the composites were exposed to the accelerated weathering conditions, some damages like cracks were formed. This primary destruction can cause an increase of material degradation.

The results of flexural strength, strain at break and the mechanical loss factor confirmed the aging of the green compositions from the unsaturated polyester resin. The other properties (glass-transition temperature, mechanical and thermomechanical) were improved with the synergistic impact of ultraviolet radiation, heat and humidity. According to Yunying and Jiagyan [21] the main factor responsible for polyester failure is UV irradiation. In the composites degradation temperature and water diffusion play important role [26,27,28]. The wood–resin composites become stiffer after ageing, which can be mainly attributed to post-curing of the material caused by temperature and UV radiation [29]. The addition of dba had a positive effect on the composite properties. Due to the fact that it absorbed UV light, it slowed down the aging process. There was no degradation of ester groups and the structure of the polyester remained stable. The presence of double bonds in dba increased the degree of resin cross-linking. Its addition protected the resin products exposed to weather conditions against aging.

Based on the obtained results, it can be concluded that the tested material with wood flour and dibenzylideneacetone can be exposed to UV radiation maintaining gloss and without significant changes of hardness, therefore it can be exposed to the external environment.

## 4. Conclusions

Green composites obtained from the unsaturated polyester resin based on the recycled PET, wood flour and polymeric cobalt as an initiator not being released from the polymeric structure were aged. With an increase in the wood flour content, the mechanical properties of the composites deteriorated. Their flexural strength, strain at break and mechanical loss factor decreased. After aging composites became brittle and their gloss diminished.

To delay the aging process of composites, dba was used. This compound, used as a UV filter, added to the unsaturated polyester resins and their composites improved significantly their aging resistance. No degradation of the polyester structure was observed. Double bonds from dba cause additional crosslinking of the resin. As a result, composites with better flexural strength, hardness and gloss were obtained.

## Figures and Tables

**Table 1:** Compositions of green composites.

Sample	UPR (wt %)	WF (wt %)	DB (wt %)
UPR	100.0	-	-
U+W1	99.0	1.0	-
U+W2	98.0	2.0	-
U+W5	95.0	5.0	-
U+d0.2	99.8	-	0.2
U+d0.5	99.5	-	0.5
U+d1.0	99.0	-	1.0
U+W5+d1.0	94.0	5.0	1.0

**Table 2:** Mechanical and thermomechanical data of the samples before accelerated aging test.

Sample	Flexural Modulus	Flexural Strength	Strain at Break	Barcol Hardness	Mechanical Loss Factor	Full Width at Half Maximum
	E_f_ (GPa)	σ_f_ (MPa)	ε (%)	HBa (°B)	tan δ_max_	FWHM (°C)
UPR	3.59 ± 0.03	108.19 ± 3.77	3.41 ± 0.06	36.0	0.4579	46.1
U+W1	3.61 ± 0.04	81.96 ± 2.71	2.59 ± 0.13	39.5	0.4419	45.9
U+W2	3.67 ± 0.03	76.83 ± 2.65	2.43 ± 0.07	40.3	0.4368	43.4
U+W5	3.81 ± 0.01	74.61 ± 1.48	2.28 ± 0.02	45.2	0.4227	42.4
U+d0.2	3.58 ± 0.01	101.96 ± 3.87	3.09 ± 0.09	48.4	0.4617	42.4
U+d0.5	3.66 ± 0.04	108.03 ± 3.92	3.22 ± 0.09	49.3	0.4621	42.0
U+d1.0	3.68 ± 0.03	120.07 ± 1.73	3.89 ± 0.05	52.0	0.4654	40.5
U+W5+d1.0	3.85 ± 0.01	73.93 ± 1.42	2.37 ± 0.02	52.1	0.4293	40.8

**Table 3:** Mechanical and thermomechanical data of the samples after the accelerated aging test.

Sample	Flexural Modulus	Flexural Strength	Strain at Break	Barcol Hardness	Mechanical Loss Factor	Full Width at Half Maximum
	E_f_ (GPa)	σ_f_ (MPa)	ε (%)	HBa (°B)	tan δ_max_	FWHM (°C)
UPR	3.78 ± 0.02	69.26 ± 4.35	2.07 ± 0.09	56.4	0.4839	39.7
U+W1	3.83 ± 0.04	50.03 ± 3.33	1.31 ± 0.08	55.7	0.4510	39.6
U+W2	3.90 ± 0.01	40.95 ± 4.27	1.32 ± 0.11	54.1	0.4397	40.0
U+W5	3.94 ± 0.03	39.43 ± 4.39	1.26 ± 0.11	51.3	0.4131	38.5
U+d0.2	3.80 ± 0.02	84.18 ± 4.36	2.70 ± 0.02	53.3	0.4630	40.7
U+d0.5	3.80 ± 0.03	84.41 ± 4.40	2.61 ± 0.09	52.5	0.4548	40.8
U+d1.0	3.79 ± 0.02	86.82 ± 4.86	2.55 ± 0.11	52.0	0.4584	41.1
U+W5+d1.0	3.93 ± 0.02	51.48 ± 4.09	1.45 ± 0.05	55.1	0.4214	39.5

**Table 4:** Glass-transition temperature and storage modulus in the glassy and rubbery regions (from the storage modulus curve) data of green composites before and after the accelerated aging test.

Sample	Glass-Transition Temperature, T_g_ (°C)				Storage Modulus, E’			
	from tan δ		from Loss Modulus Curve		Glassy, E’ (20 °C) (GPa)		Rubbery, E’ (180 °C) (MPa)	
	Before	After	Before	After	Before	After	Before	After
UPR	127	132	100	107	2.91	2.81	24.06	25.24
U+W1	126	131	101	108	3.17	2.78	31.45	23.78
U+W2	126	131	103	108	2.94	2.81	34.68	28.50
U+W5	126	130	105	110	2.86	2.90	49.08	40.04
U+d0.2	127	132	105	109	2.91	2.90	27.92	23.37
U+d0.5	127	131	105	109	2.90	2.88	26.51	23.84
U+d1.0	128	131	105	108	2.92	2.87	23.51	22.69
U+W5+d1.0	126	130	106	109	2.96	2.90	41.68	33.58

**Table 5:** Gloss measurement data of green composites before and after the accelerated aging test.

Sample	Gloss (GU)					
	20°	60°	85°	20°	60°	85°
	Before Aging			After Aging		
UPR	101.3	112.4	100.4	30.6	50.2	81.1
U+W1	96.8	110.4	98.5	63.3	92.2	96.0
U+W2	96.4	101.8	99.2	57.5	84.0	90.3
U+W5	76.8	95.3	96.1	55.3	81.0	87.6
U+d1.0	133.3	128.7	105.5	89.3	98.8	98.3
U+W5+d1.0	94.1	100.6	100.8	77.6	91.6	96.5
